# Oral infectivity through carnivorism in murine model of *Trypanosoma cruzi* infection

**DOI:** 10.3389/fcimb.2024.1297099

**Published:** 2024-02-22

**Authors:** Víctor Torres, Víctor Contreras, Bessy Gutiérrez, Juan San Francisco, Alejandro Catalán, José Luis Vega, Kyung-Mee Moon, Leonard J. Foster, Rafael F. de Almeida, Alexis M. Kalergis, Jorge González

**Affiliations:** ^1^ Molecular Parasitology Unit, Medical Technology Department, University of Antofagasta, Antofagasta, Chile; ^2^ Centro de Salud El Salvador, El Salvador, Chile; ^3^ Departamento de Fisiologia, Facultad de Ciencias Biológicas, Universidad de Concepción, Concepción, Chile; ^4^ Department of Biochemistry & Molecular Biology, Michael Smith Laboratories, University of British Columbia, Vancouver, BC, Canada; ^5^ Laboratório de Biologia Molecular e Sistêmica de Tripanossomatídeos (Labtryp), Instituto Carlos Chagas Fiocruz (ICC-Fiocruz), Curitiba, Paraná, Brazil; ^6^ Millennium Institute of Immunology and Immunotherapy, Facultad de Ciencias Biológicas, Pontificia Universidad Católica de Chile, Santiago, Chile; ^7^ Departamento de Endocrinología, Facultad de Medicina, Pontificia Universidad Católica de Chile, Santiago, Chile; ^8^ Research Center in Immunology and Biomedical Biotechnology of Antofagasta, Universidad de Antofagasta, Antofagasta, Chile

**Keywords:** *Trypanosoma cruzi*, oral infection, cruzipain, trans-sialidase, host-pathogen interaction

## Abstract

**Introduction:**

Oral transmission of *T. cruzi* is probably the most frequent transmission mechanism in wild animals. This observation led to the hypothesis that consuming raw or undercooked meat from animals infected with *T. cruzi* may be responsible for transmitting the infection. Therefore, the general objective of this study was to investigate host-pathogen interactions between the parasite and gastric mucosa and the role of meat consumption from infected animals in the oral transmission of *T. cruzi.*

**Methods:**

Cell infectivity assays were performed on AGS cells in the presence or absence of mucin, and the roles of pepsin and acidic pH were determined. Moreover, groups of five female Balb/c mice were fed with muscle tissue obtained from mice in the acute phase of infection by the clone H510 C8C3 ***hvir*** of *T. cruzi*, and the infection of the fed mice was monitored by a parasitemia curve. Similarly, we assessed the infective capacity of *T. cruzi* trypomastigotes and amastigotes by infecting groups of five mice Balb/c females, which were infected orally using a nasogastric probe, and the infection was monitored by a parasitemia curve. Finally, different trypomastigote and amastigote inoculums were used to determine their infective capacities. Adhesion assays of *T. cruzi* proteins to AGS stomach cells were performed, and the adhered proteins were detected by western blotting using monoclonal or polyclonal antibodies and by LC-MS/MS and bioinformatics analysis.

**Results:**

Trypomastigote migration in the presence of mucin was reduced by approximately 30%, whereas in the presence of mucin and pepsin at pH 3.5, only a small proportion of parasites were able to migrate (∼6%). Similarly, the ability of TCTs to infect AGS cells in the presence of mucin is reduced by approximately 20%. In all cases, 60–100% of the animals were fed meat from mice infected in the acute phase or infected with trypomastigotes or amastigotes developed high parasitemia, and 80% died around day 40 post-infection. The adhesion assay showed that cruzipain is a molecule of trypomastigotes and amastigotes that binds to AGS cells. LC-MS/MS and bioinformatics analysis, also confirmed that transialidase, cysteine proteinases, and gp63 may be involved in TCTs attachment or invasion of human stomach cells because they can potentially interact with different proteins in the human stomach mucosa. In addition, several human gastric mucins have cysteine protease cleavage sites.

**Discussion:**

Then, under our experimental conditions, consuming meat from infected animals in the acute phase allows the *T. cruzi* infection. Similarly, trypomastigotes and amastigotes could infect mice when administered orally, whereas cysteinyl proteinases and trans-sialidase appear to be relevant molecules in this infective process.

## Introduction

Chagas disease, caused by the protozoan *Trypanosoma cruzi*, affects 6–7 million people worldwide, with an annual incidence of 28 thousand cases in the Americas (PAHO/WHO, 2022). Human infections occur via fecal feces released by contaminated insect vectors belonging to the Reduviidae family, blood transfusion, vertical transmission, organ transplantation, laboratory accidents, and oral infections (Schmunis and Yadon, 2010; Sánchez and Ramírez, 2013). As a result of various initiatives aimed at controlling vector transmission and eradicating household vectors, the incidence of Chagas disease has been reduced in recent years (Coura and Vĩas, 2010; Rassi et al., 2010; Coura, 2015). Similarly, the massive implementation of serological screening techniques in blood banks in endemic regions has significantly reduced post-transfusion transmission of Chagas disease (Rassi et al., 2010; de Arias et al., 2022). However, in recent decades, oral transmission as a result of the ingestion of contaminated food and beverages as a result of liquefaction of whole triatomines or contamination with feces from infected triatomines has begun to occupy a relevant place in the mechanisms of transmission of Chagas disease (Coura, 2006; Coura, 2015; de Noya and González, 2015). Currently, oral transmission of *T. cruzi* is the most significant transmission route in Brazil (70–80% of cases) (Coura, 2006). In addition, some outbreaks have been described in several South American countries, such as Venezuela, Colombia, Bolivia, Argentina, French Guyana and Ecuador (Coura et al., 1997; Benchimol Barbosa, 2006; Dias et al., 2008; De Noya et al., 2010; Shikanai-Yasuda and Carvalho, 2012; Ramírez et al., 2013; Sánchez and Ramírez, 2013). These outbreaks were associated with consuming contaminated food and beverages such as wild animal meat, vegetables, sugar cane extract, açaí pulp, guava juice, bacaba, babaçu, and palma wine (Ferreira et al., 2001; Pereira et al., 2009; Barbosa-Ferreira et al., 2010). Furthermore, from 1968 to 2000, 50% of acute cases in the Amazon region were attributed to oral transmission (Shikanai-Yasuda and Carvalho, 2012; Bruneto et al., 2021), and these numbers later reached 70% between 2000 and 2010 (Shikanai-Yasuda and Carvalho, 2012). Venezuela has also reported the most significant outbreak described to date, with two distinct occurrences affecting 103 and 88 people, respectively. These outbreaks affect adults and children from urban and rural schools (De Noya et al., 2010; Sánchez and Ramírez, 2013). The mortality rate of orally infected patients is higher (0–44%) than that of classical vectorial transmission through triatomine fecal contamination after biting (<5–10%) (López-García and Gilabert, 2023). Oral transmission is currently the most frequent form of transmission (Coura, 2015).

Many observations have suggested that carnivorism may play a central role in *T. cruzi* transmission, especially in wildlife, when predators devorate their prey (Coura et al., 2002; Coura, 2006). However, this hypothesis has not been experimentally demonstrated.

In this study, we demonstrate that consuming raw meat transmits the oral infection by *T. cruzi*, and both trypomastigote and amastigote stages are infective for mice when administered by a nasogastric tube. Additionally, cysteinyl proteinases (among them, cruzipain) and trans-sialidase (TS) appear to be involved in parasite adhesion or invasion of gastric cells since both molecules are associated with AGS stomach cells.

## Material and methods

### Parasite cell lines and cell culture

The virulent *T. cruzi* cell line, C8C3 ***hvir***, and low-virulence C8C3 ***lvir*** were obtained from clone H510 C8C3, as previously described (San Francisco et al., 2017). Vero cells were infected with tissue culture-derived trypomastigotes (TCTs) as described previously (Araya et al., 2008). Five days later, the supernatants containing more than 95% TCT were collected by centrifugation.

### Trypomastigote migration assay using gastric mucin layer

These tests were performed as previously described (Cortez et al., 2012). Briefly, polycabonate transwell filters (3 μm pores, diameter) were coated with 50 μl of a solution of gastric mucin dissolved in Dulbecco’s Modified Eagle’s Medium (DMEM) (10 mg/mL) and the migration of TCT (1x10^7^/well) was assayed over a period of 1 h.

### Trypomastigote treatment with pepsin

Trypomastigotes were treatment or not with pepsin as previously described (Cortez et al., 2012). Briefly, TCTs, were incubated for 30 minutes in a solution of pepsin (2 mg/mL) dissolved in DMEM medium adjusted to pH 3.5. At the end of the incubation period, the number of trypomastigotes was determined by using a Neubauer chamber. In another set of experiments, trypomastigotes treated or not with pepsin for 30 min were lysed with PBS plus 0.1% NP-40 containing cOmplete^™^ Mini EDTA-free Protease Inhibitor Cocktail (Thermo Fisher Scientific, Rockford, IL, USA) and subjected to sodium dodecyl sulfate-polyacrylamide gel electrophoresis. ImageJ v2.0 software was used to perform densitometric analysis of the gels,. To obtain consistent results, each gel was repeated at least three times.

### *In vitro* invasion assay in the presence or absence of mucin

AGS cells (ATCC CRL-1739), a cell line isolated from human gastric adenocarcinoma from stomach tissue, were seeded at 5 × 10^4^ cells/well in 4-well Lab-Tek Chamber Slides (NUNC, Thermo Fisher Scientific, Roskilde, Denmark). The TCT invasion assay was performed according to a previously reported method using a C8C3 ***hvir*** and C8C3 ***lvir*** at a parasite:cell ratio equal to 10:1 (Araya et al., 2008). Parasites were then placed in contact with the cells in the absence or presence of 500 μg mL^-1^ of gastric mucin from the porcine stomach (type III; Sigma), as previously reported (Neira et al., 2003). The cells were incubated for 3 h at 37°C. The cells were washed three times with phosphate-buffered saline (PBS), fixed with methanol, and stained with 4′,6-diamidino-2-fenilindol (DAPI). The number of parasites in 500 cells was determined using a BX51 fluorescence microscope (Olympus Corporation, Tokyo, Japan). Images of *T. cruzi*-infected cells were obtained by confocal immunofluorescence microscopy (Leica TCS SP8 microscope, Leica Microsystems GmbH, Wetzlar, Alemania).

### *In vivo* infection and parasitemia curve

Four- to six-week-old female BALB/c mice were infected with TCTs from C8C3*hvir* cell line through intraperitoneal injection (1×10^5^ parasites per mouse). Parasitemia was monitored every two days starting on day 2 post-inoculation, as previously described (Brener, 1962), and experiments were stopped on day 20, near peak parasitemia. The animals were euthanized, and the muscles were dissected and used to feed female mice, as previously described (Rani et al., 2019). In these experiments, each animal was fed infected mouse meat and placed in an individual cage to control the experiment and avoid competition for the food source, thus guaranteeing total intake of the infected meat. The mice that ingested the meat of the infected animals had the same characteristics, and in the last 24 h, they received water *ad libitum* with solid food deprivation. Parasitemia was evaluated from the second day onwards, as described above.

For oral infection, four to six-week-old female Balb/c mice were infected with *T. cruzi* TCTs or amastigotes via the oral route (1 × 10^5^ parasites per mouse) using a nasopharyngeal probe adapted to a 1 ml syringe. Starting on day 5 post-inoculation, parasitemia was monitored every two days, twice a week, by examining 5 µL blood samples collected from the tail and observed under a phase contrast microscope (Staquicini et al., 2010). In all cases, gastric mucosal infection was monitored by qPCR, where total genomic DNA was extracted from stomach of the infected mice using the Wizard Genomic DNA Purification Kit (Promega) according to the manufacturer’s instructions. DNA concentration was quantified using an Infinite 200 PRO UV spectrophotometer (Tecan, Männedorf, Switzerland) and adjusted to 25 ng/μL as previously described (San Francisco et al., 2022).

To assess the potential role of cysteinyl proteinases in parasite-host interactions during the oral route, TCTs were treated with (2S,3S)-*trans*-Epoxysuccinyl-L-leucylamido-3-methylbutane ethyl ester (E64d) as previously described (San Francisco et al., 2017). Briefly, TCTs were incubated for 45 min at room temperature with 50 µM of the cell-permeable cysteinyl proteinases inhibitor, E64d. At the end of this incubation period, parasite viability was assessed by motility and membrane integrity was evaluated by propidium iodide staining. Subsequently, the TCTs were washed and used for oral infections using a nasopharyngeal probe.

All experiments were conducted according to the Institutional Ethical Committee for Animal Experimentation regulations, and all protocols were approved by them (CEIC REV/2013).

### SDS-PAGE and immunoblots

TCTs or amastigotes from the C8C3 ***hvir** T. cruzi* cell line were lysed by sonication (PBS 1X containing cOmplete™, Mini, EDTA-free Protease Inhibitor Cocktail) (Thermo Fisher, Rockford, IL, USA), and protein concentration was determined (Bradford, 1976). Then, soluble extracts of TCT or amastigotes were incubated with AGS cell cultures at 4°C for 4 h. Finally, the cell culture medium was removed, and the cultures were washed three times with PBS. The cultures were lysed (NP-40 1%, PBS 1X containing cOmplete™, Mini, EDTA-free Protease Inhibitor Cocktail) (Thermo Fisher, Rockford IL, USA) and the soluble extract was analyzed by SDS-PAGE on a 12% acrylamide gel and electrotransferred to nitrocellulose membranes in a Trans-Blot Turbo (Bio-Rad Hercules CA, USA). Immunoreactivity was determined by incubation with a polyclonal antibody against cruzipain (Czp) (either prepared by us (JG) or provided by Dr. Juan José Cazzulo of the Universidad Nacional de San Martin, Buenos Aires, Argentina); mouse anti-recombinant complement regulatory protein (CRP) antibody (provided by Dr. Karen Norris of Department of Infectious Diseases in the College of Veterinary Medicine, University of Georgia, Athens, USA), and monoclonal antibody 39 against trans-sialidase (TS) (provided by Dr. Sergio Schenkman of the Federal University of Sao Paulo, Brazil), monoclonal antibody 2C2 against Ssp4 amastigote antigen (amastin) (Andrews et al., 1987). Anti-rabbit or anti-mouse IgG peroxidase-labeled antibodies were used as secondary antibodies. Immunoblots were developed using enhanced chemiluminescence assay (ECL), (Thermo Fisher, Rockford, IL, USA). Using the Gel-Capture software, ECL Images were captured in a ChemiDoc MP Imaging System (BioRad, Hercules, CA, USA).

### Membrane shaving and in-gel protein digestion

AGS cells were incubated with soluble extracts of *T. cruzi* TCT from the C8C3 ***hvir*** cell line for 4h at 4°C as described above. The cultures were washed three times with PBS, and the cells were trypsinized. The cells and trypsinized material were collected and centrifuged at 1800 rpm, and the supernatant containing the peptides obtained by shaving was concentrated using a Speed Vac (Rees et al., 2015). In-gel protein digestion was performed as described by San Francisco et al. (2022).

### Reverse phase-liquid chromatography-tandem mass spectrometry (RP- LC-MS/MS) analysis

Peptide concentrations were measured on NanoDrop One (Thermo Fisher - A205, scopes) to load approximately 50 ng of peptides on TimsTOF Pro2 (Bruker Daltonics) with CaptiveSpray source coupled to nanoElute UHPLC (Bruker Daltonics) using Bruker’s PepSep Ten column (10 cm x 75 µm inner diameter, 1.9 µm particle size with 20 µm emitter) heated to 40°C. Buffer A consisted of 0.1% formic acid and 0.5% acetonitrile in water, and buffer B consisted of 0.1% formic acid and 99.4% acetonitrile in water. A standard 30 min gradient was used, increasing buffer B percentages from 2% to 12% over 15 min and then to 33% over 15 min. The column was washed with 95% B over 8 min. The NanoElute thermostat temperature was set at 7°C. The analysis was performed at a 0.4 μL/min flow rate.

The TimsTOF Pro2 was set to DIA-PASEF mode with positive polarity for the MS scan window from 100 to 1700 m/z. The capillary voltage was set to 1800V, drying gas to 3L/min, and drying temperature to 180°C. The MS1 scan was followed by PASEF ramps containing 22 non-overlapping 35 m/z isolation windows, covering the mass range 319.5 – 1089.5 Da. Ion mobility range (1/K0) was set to 0.70 – 1.35 V·s/cm2, with 100 ms ramp time, 100 ms accumulation time, 100% duty cycle, and 9.42 Hz ramp rate. The collision energy was ramped linearly as a mobility function from 27eV at 1/K0 = 0.70 V·s/cm2 to 55eV at 1/K0 = 1.35 V·s/cm2.

The acquired data were searched with DIA-NN (PMID31768060) version 1.8.1 with open access library search method, using UniProt’s human-reviewed protein sequences, three *Trypanosoma cruzi* reference proteomes (UP000002296, UP000246078, UP000246121), and manually curated common contaminants (226 entries). Other search parameters include Trypsin/P digestion mode with 1 missed cleavage, 1 maximum number of variable modification, N-terminal M excision, carbamidomethylation of C, and oxidation of M options enabled, peptide length ranged 7-30, precursor charge ranged 1-4, precursor m/z ranged 300-1800, and fragment ion m/z ranged 200-1800. Precursor False Discovery Rate (FDR) was set to 1%, with 0 Mass accuracy and MS1 accuracy (for the “auto” option of mass tolerance), enabling heuristic protein inference, using isotopologues, match between run (MBR), and no shared spectra. Protein inference is set as “Protein name from FASTA”, Double-pass mode for neural network classifier, Robust LC (high precision) for Quantification Strategy, RT-dependent mode for Cross-run normalization, and Smart profiling mode for Library generation.

The mass spectrometry proteomics data have been deposited to the ProteomeXchange Consortium via the PRIDE (PubMed ID: 34723319) partner repository with the dataset identifier PXD044267.

### Protein-protein interactions analysis

Protein-protein interactions (PPIs) were mapped and visualized using the Search Tool for the Retrieval of Interacting Genes/Proteins (STRING) (Doncheva et al., 2023) in Cytoscape v.3.10 (Shannon et al., 2003) using StringApp 2.0 (Doncheva et al., 2023) to retrieve the cross-species network. First, a full-string network was generated with the PPIs of *Homo sapiens* and *T. cruzi* with a confidence score cutoff of ≥ 0.4. The PPIs involving membrane-related *T. cruzi* proteins and human proteins identified by LC-MS/MS in this study and their neighbors up to one level of interaction were mapped into the networks. The maps were drawn using the Cytoscape software. Next, we analyzed gene–tissue associations of human proteins mapped in the interaction networks to identify their scores in the stomach tissue. For this purpose, TISSUE 2.0.database (Palasca et al., 2018) integrated into Cytoscape StringApp was used, and the proteins were ranked according to the expression score in that tissue.

### Prediction of cysteine protease cleavage sites

The cysteine protease cleavage sites were predicted using GPS-CCD 1.0 tool (Liu et al., 2011) with a high cut-off value (≥ 0.654). The top 10 scored cleavage sites are shown for each mucin mapped in the interaction analysis using DOG 2.0 (Ren et al., 2009). These domains were identified using the conserved domain database (CDD-NCBI) (Wang et al., 2023) and the specific hits added to the DOG 2.0. Next, the sequence window (a total of 10 amino acids) of the predicted cleavage sites were analyzed to map the amino acid motifs upstream to the cleavage site. The kpLogo tool was used, and the single amino acid motifs calculated as the most significant were visualized as k-mer logos.

### Statistical analyses

Statistical analysis was performed using Prism 5 software (GraphPad Software) using Student’s t-test and analysis of variance (ANOVA). The significance of the *T. cruzi* experiments, in which differences in infectivity or cell invasion between the C8C3 ***hvir*** and C8C3 ***lvir*** cell lines were compared. Statistical significance was set at p<0.01, which was considered significant.

## Results

**Figure 1:**
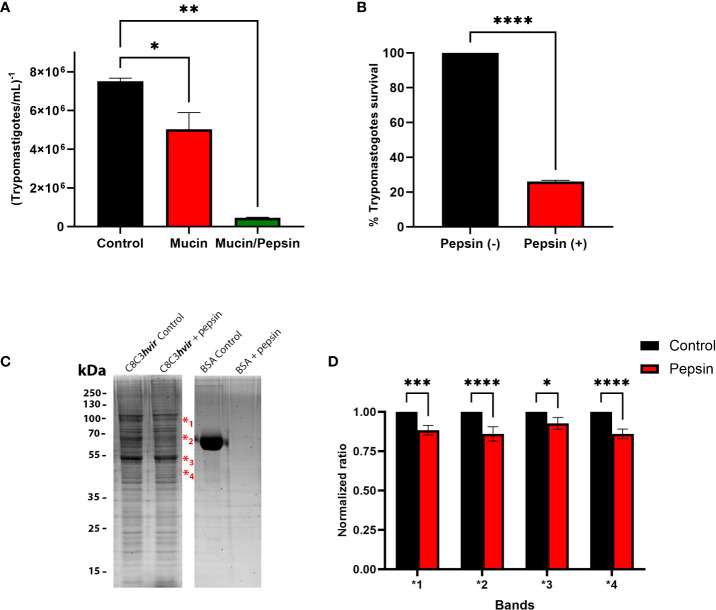
Evaluation of *Trypanosoma cruzi* trypomastigote migration through gastric mucin. **(A)** Gastric mucin was deposited on transwell chamber filters. After 30 min, a suspension of tryponastigotes (1x10^7^) was added and incubated for 1 h in the presence or absence of pepsin at pH 3.5. The number of trypomastigotes that migrated after 1 h of incubation was determined using the Neubauer chamber count and compared with controls, in which gastric mucin was absent. **(B)** Trypomastigotes were treated or untreated with pepsin for 30 min and at the end of the incubation period were counted in a Neubauer chamber and the percentage of survival was determined. **(C)** Trypomastigotes treated or untreated with pepsin for 30 min were lysed and subjected to 12% sodium dodecyl sulfate-polyacrylamide gel electrophoresis. The gels were stained with Sypro Ruby. **(D)** Densitometric analysis of gel were performed using imageJ 2.0v software, where each band that showed a decrease in intensity after treatment of the parasites with pepsin was compared with the homologous band of the untreated control. Bars are represented as the mean ± SEM of the least three independent experiments. ***P < 0.0002 (band 1); *P < 0.0127; ****P < 0.0001 (band 2 and 4) vs corresponding control; Two way anova. ** p=0.0016.

The ability of TCTs to traverse a mucin layer was evaluated in microtiter plates with transwell filters coated with gastric mucin, by counting the number of TCTs traslocated through the mucin layer after 30 minutes incubation. As shown in **Figure 1A**, when migration experiments in the presence or absence of mucin were performed, the migration of TCTs was reduced by approximately 30% in the presence of mucin (**Figure 1A**). An even more significant reduction in migration was observed in migration experiments in the presence of mucin and pepsin at pH 3.5. In these cases, only a small number of parasites (∼ 6%) had migrated (**Figure 1A**). To examine the susceptibility of TCTs to pepsin, they were incubated for 30 min in DMEM medium pH 3.5 containing pepsin (2 mg/mL^-1^). TCTs were highly sensitive to pepsin lysis, and only 25.5% of C8C3*hvir* parasites survived (**Figure 1B**). To gain preliminary insights into the effect of pepsin on the surface proteins of TCTs, an SDS-PAGE gel was developed. As shown in **Figure 1C**, expression of at least four bands between 40 and 90 kDa was reduced after pepsin treatment, suggesting that they could probably be digested by pepsin. These differences were also visualized in the densitometric analysis, where statistically significant differences were observed in the intensity of the bands in the pepsin-treated parasite run with respect to the controls that did not receive this treatment (**Figure 1D**).

**Figure 2:**
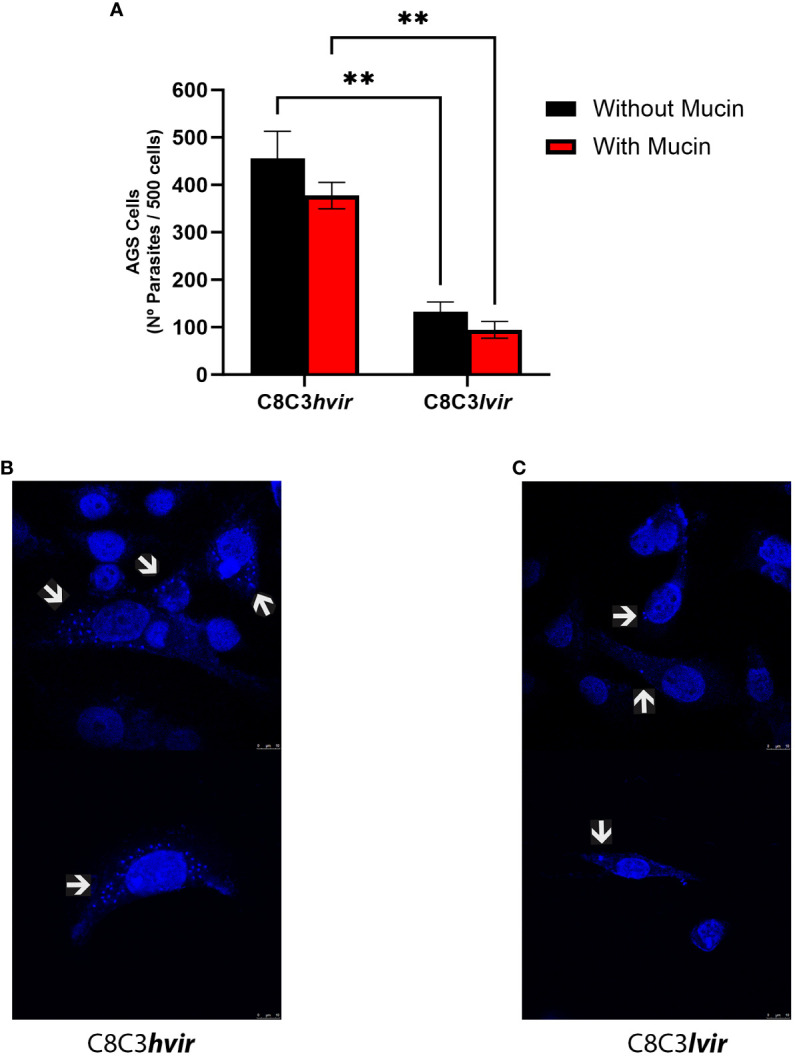
*In vitro* infectivity of *Trypanosoma cruzi* C8C3*hvir* and C8C3*lvir* cell lines. **(A)** AGS cells were infected with trypomastigotes from either C8C3 ***hvir*** or C8C3 ***lvir*** cell lines, in presence or absence of mucin, using a parasite/cell ratio 10:1. After 3h, cultures were washed three times with phosphate-buffered saline and stained with 4′,6-diamidino-2-fenilindol. Results are expressed as the mean ± SD of parasites/500 cells from three experiments performed in triplicate. Representative images of the infectivity of the C8C3 ***hvir*** **(B)** and C8C3 ***lvir*** **(C)** T.cruzi cell lines for AGS cells were acquired using a Leica TCS SP8 confocal fluorescence microscope with a 700 Hz gain and a DMOD direct intensity modulated solid-state violet laser. Without mucin ** p=0.0066; With mucin ** p=0.0087.

Next, we evaluated the capacity of TCTs to invade AGS human stomach cells in the presence or absence of gastric mucin, in an attempt to mimic the *in vivo* situation in which trypomastigotes interact with and traverse the mucous layer before coming into contact with stomach epithelial cells. An invasion assay using two *T. cruzi* cell lines (high virulence and low virulence) showed that both *T. cruzi*, C8C3 ***hvir*** and C8C3 ***lvir*** could invade AGS cells. However, the C8C3 ***hvir*** cell line was at least three times more invasive than C8C3 ***lvir***. Therefore, it was selected for experimental studies in mice. In the experiments in which mucin was present, a decrease of approximately 20% in the invasiveness of both cell lines was observed, as reflected by the lower number of intracellular parasites after 3 h of incubation compared to that observed in the absence of mucin (**Figure 2A**). As shown in **Figure 2B**, AGS cells were highly infective for TCTs of the virulent cell line (C8C3 ***hvir***), while the low virulence cell line (C8C3 ***lvir***) was poorly infective against these cells (**Figures 2B, C**).

**Figure 3:**
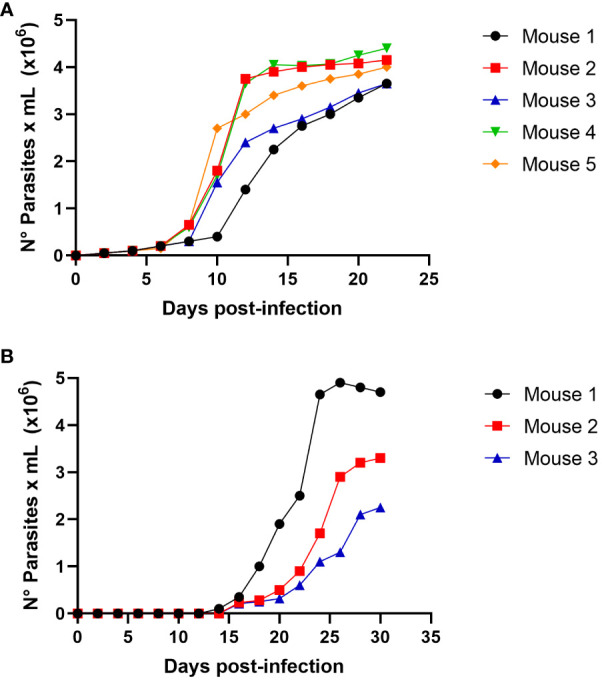
*Trypanosoma cruzi* oral infection by mice carnivorism **(A)** Groups of 5 female Balb/c mice were infected intraperitoneally with 1x10^5^ trypomastigotes derived from cell culture of *T.cruzi* C8C3 ***hvir*** cell line. Parasitemia curve was determined by counting (every 2 days) the number of parasites in a blood sample obtained through a small cut in the tail of the mouse. **(B)** Groups of 5 female Balb/c mice were fed with mice muscles obtained at the mice acute infection phase, as shown in Figure **(A)**. The appearance and evolution of parasitemia was monitored as described in **(A)**. Results are expressed as mean ± 1 SD and represent at least 3 experiments performed with the same protocols.

To evaluate the possibility of *T. cruzi* transmission by carnivorism, we initially infected mice intraperitoneally, which were sacrificed in the acute phase at the peak of parasitemia (**Figure 3A**). The sacrificed animals were dissected, and raw meat was given as food to uninfected mice that had been starved for two days. Oral infection with *T. cruzi* in animals was monitored using parasitemia curve analysis. As shown in **Figure 3B**, three of the five mice (60%) were infected with *T. cruzi* and died due to the infection.

**Figure 4:**
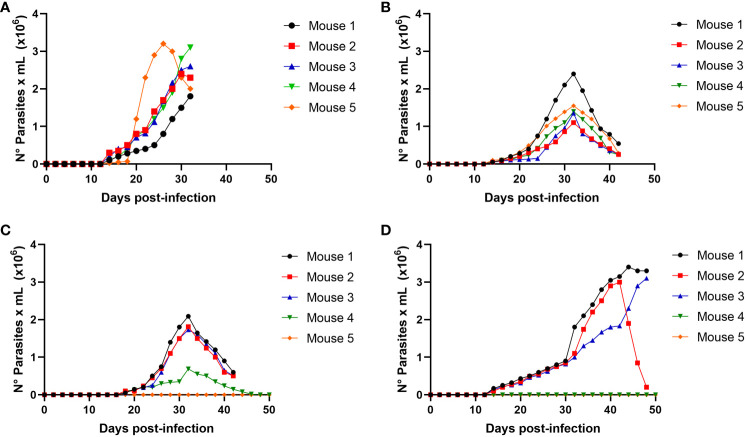
Oral infectivity of *Trypanosoma cruzi* amastigotes. Groups of five female Balb/c mice were orally infected using a nasogastric probe with 4 × 10^5^ **(A)**, 3 × 10^5^ **(B)**, 2 × 10^5^ **(C)**, and 1 × 10^5^ **(D)** *T. cruzi* amastigotes from the C8C3*hvir* cell line. The parasitemia curve was determined by counting (every 2 days) the number of parasites in a blood sample obtained from a small cut in the tail of the mouse. Results are expressed as mean ± 1 SD and represent at least 3 experiments performed with the same protocols.

**Figure 5:**
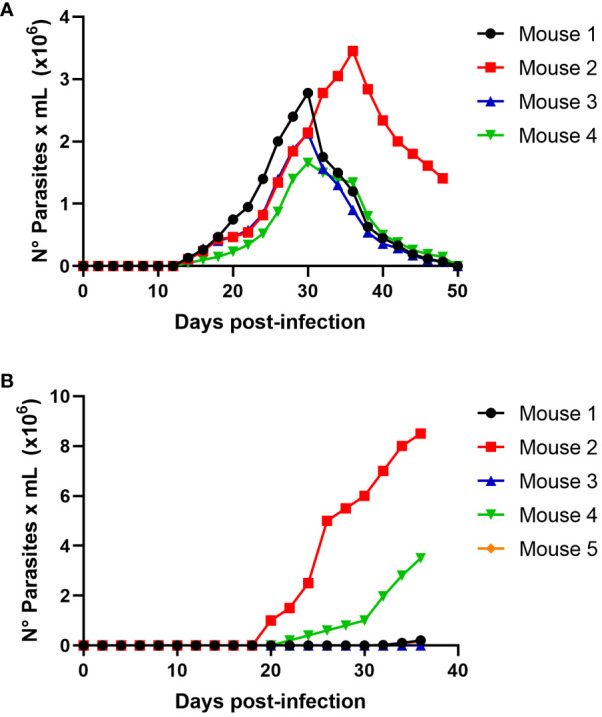
Oral infectivity of *Trypanosoma cruzi* trypomastigotes. Groups of five female Balb/c mice were orally infected using a nasogastric probe with × 4x10^5^ **(A)** and 1 × 10^5^ **(B)** *T. cruzi* trypomastigotes from the C8C3*hvir* cell line. Parasitemia curve was determined by counting (every 2 days) the number of parasites in a blood sample obtained through a small cut in the tail of the mouse. Results are expressed as the mean ± 1 SD and represent at least three experiments performed using the same protocols.

We determined whether the infection produced by raw meat from the infected mice resulted from trypomastigotes or intracellular amastigotes. Then, experimental infections were performed using a nasogastric probe and different parasite stages and inoculums. When a group of mice was experimentally infected with different amastigotes inocula using a nasogastric probe, all animals were infected (**Figure 4**). One hundred % of mice were infected with an inoculum of 3 × 10^5^ cells (**Figure 4B**), whereas 80% were infected with an inoculum of 2 × 10^5^ cells (**Figure 4C**). Moreover, 60% of the mice were infected with the inoculum of 1x10^5^ (**Figure 4D**). Also, as shown in **Figure 5A**, trypomastigotes were infectives when inoculums of 4× 10^5^ cells were used, resulting in four of five infected mice (80%). When inoculums of 1×10^5^ were used, 4 of 5 mice inoculated (80%) were also infected (**Figure 5B**). It is well known t It is well known that the route of parasite administration is critical for the course of infection, with different courses of parasitemia and mortality. Thus, inoculation in the mouth (oral gavage) results in higher levels of parasitemia and mortality than intragastric or nasogastric infections (Barreto-de-Albuquerque et al., 2015). In oral infection experiments with TCTs or amastigotes, we preferred to use a nasogastric tube instead of oral infection. We chose this procedure for two reasons. First, since the objective was to evaluate the infectivity of TCTs and amastigotes, we decided to determine the infective capacity of the gastric mucosa, avoiding contact of the parasites with the oral mucosa or upper digestive tract. Second, we tried to avoid premature mortality in infected mice.

**Figure 6:**
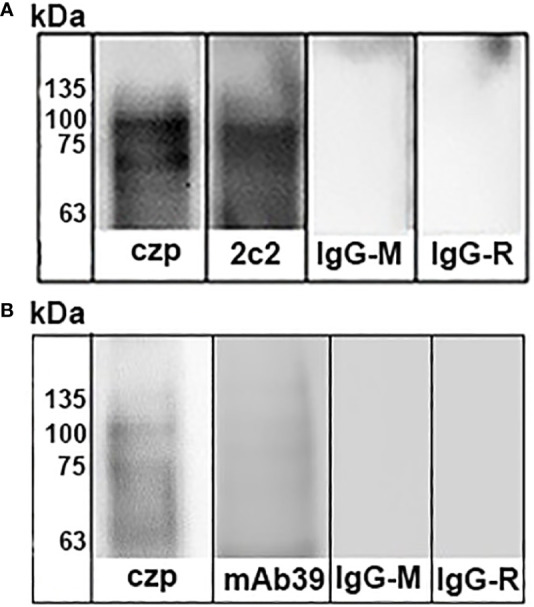
Identification of *Trypanosoma cruzi* binding to stomach cells. *T. cruzi* trypomastigotes **(A)** or amastigotes **(B)** were lysed by sonication and then centrifuged at 12.000 rpm for 10 min. Supernatants were incubated in flasks containing AGS cells for 4 h at 4°C. The cultures were washed four times and lysed with detergent Nonidet P-40. Lysates were centrifuged at 12.000 rpm for 10 min, and the supernatants were subjected to SDS-PAGE and transferred to nitrocellulose membranes. Immunoblots were then incubated with antibodies raised against cruzipain (Lane 1) and Ssp-4 epitope (Lane 2). b) Trypomatigote Immunoblots were incubated with antibodies raised against cruzipain (Lane 1) and trans-sialidase (Lane 2). Finally, the blots were incubated with secondary antibodies IgG-horseradish labeled and developed using enhanced chemiluminescence.

Next, we evaluated the virulence molecules that could be involved in parasite binding to mammalian stomach cells. To do this, we used trypomastigote or amastigote cell lysates of a recognized highly virulent *T. cruzi* cell line, C8C3 ***hvir***, which expresses the main virulence factors described in *T. cruzi*. Thus, blots of human stomach AGS cells incubated with amastigote cell lysates were probed with different antibodies, and only antibodies raised against cruzipain and the ssp4 epitope, recognized by the monoclonal antibody 2C2, were detected (**Figure 6A**). In trials with trypomastigote lysates, we observed that only antibodies against cruzipain and transialidase were able to detect the proteins associated with AGS cells (**Figure 6B**). In contrast, CRP or gp85 were not detected by immunoblot assays (Data not shown).

To deepen our knowledge of the interaction of TCTs with human stomach cells, the experimental shaving approach and subsequent protein identification by LC/MS-MS showed 33 proteins that would eventually interact during the *T. cruzi* adhesion/invasion process. Considering that the AGS cell cultures were incubated with the TCTs lysate, it is possible that the proteins located in the cytoplasm and organelles are not likely to participate in the invasive processes. Therefore, considering only parasite membrane proteins, it was possible to identify trans-sialidases, cysteinyl proteinases, and Gp63 (**Table 1**).

**Table 1:** *Trypanosoma cruzi* trypomastigotes proteins identified after incubation of parasite with human gastric adenocarcinoma from stomach cells.

N°	Protein ID	Protein Name	Subcellular Location	MW (kDA)	Length
1	A0A1P8CYY9	Surface membrane protein	Membrane	42	390
2	A0A2V2UFQ4	Trans-sialidase, Group V	Membrane	82,4	772
3	A0A2V2UIH3	Glutamate dehydrogenase	Mitochondria	45,1	416
4	A0A2V2UG57	Tubulin beta chain	Microtubules	48,6	436
5	A0A2V2UJL9	Rab-GAP TBC domain-containing protein	Lysosome - secreted	57,1	500
6	A0A2V2UNL4	Superoxide dismutase	Mitochondria & cytoplasm	23,3	207
7	A0A2V2UP99	Trans-sialidase, Group II	Membrane	90,3	841
8	A0A2V2UPW0	Gp-63	Membrane	76,5	697
9	A0A2V2UGX4	Trans-sialidase, Group II	Membrane	80,6	750
10	A0A2V2UU72	Flagellar attachment zone protein 1	Flagellar	18,8	165
11	A0A2V2UUK8	Cysteine peptidase, Clan CA, family C51	Membrane	37,6	329
12	A0A2V2UWM9	Retrotransposon hot spot (RHS) protein	Telomeres	67,8	592
13	A0A2V2UIK4	Trans-sialidase, Group II	Membrane	85,7	795
14	A0A2V2V1S2	TOG domain-containing protein	Flagellar	234,3	2131
15	A0A2V2V8F3	Translation initiation factor IF-2	Ribosomes	62,8	573
16	A0A2V2V8V3	Mitochondrial outer membrane protein porin	Mitochondria	29,6	272
17	A0A2V2VAC1	FYVE-type domain-containing protein	Membrane	71,2	654
18	A0A2V2VAS5	Trans-sialidase, Group II	Membrane	82,1	749
19	A0A2V2VCE4	Triosephosphate isomerase	Cytoplasm/glycosome/nucleus/Kinetoplast	27,3	251
20	A0A2V2VFL0	Rieske iron-sulfur protein, mitochondrial	Mitochondria & cytoplasm	33,7	297
21	A0A2V2VJW8	Reticulon-like protein	RE membrane	21	186
22	A0A2V2VLE6	Intraflagellar transport protein C3	Flagellar	65,3	566
23	A0A2V2VMT5	Retrotransposon hot spot (RHS) protein	Telomeres	112,4	993
24	A0A2V2VPD4	Serine carboxypeptidase S28	Lysosome	48,8	432
25	A0A2V2VSJ9	Trans-sialidase, Group V	Membrane	98,7	922
26	A0A2V2VTI7	Uncharacterized protein	Unknown	50	423
27	A0A2V2VYA0	Uncharacterized protein	Unknown	86,2	774
28	A0A2V2VYS6	DUF726 domain-containing protein	Membrane	160	1469
29	A0A2V2W1J8	Gim5A protein	Peroxisome membrane	26,6	244
30	A0A2V2W4V1	Trans-sialidase	Membrane	94,5	889
31	A0A2V2WIL7	Leucine-rich repeat protein (LRRP)	Flagellar	231,1	2138
32	A0A2V2WNP3	Retrotransposon hot spot (RHS) protein	Telomeres	28	244
33	A0A2V2WNQ1	Prefoldin subunit 2	Unknown	16,2	148

**Figure 7:**
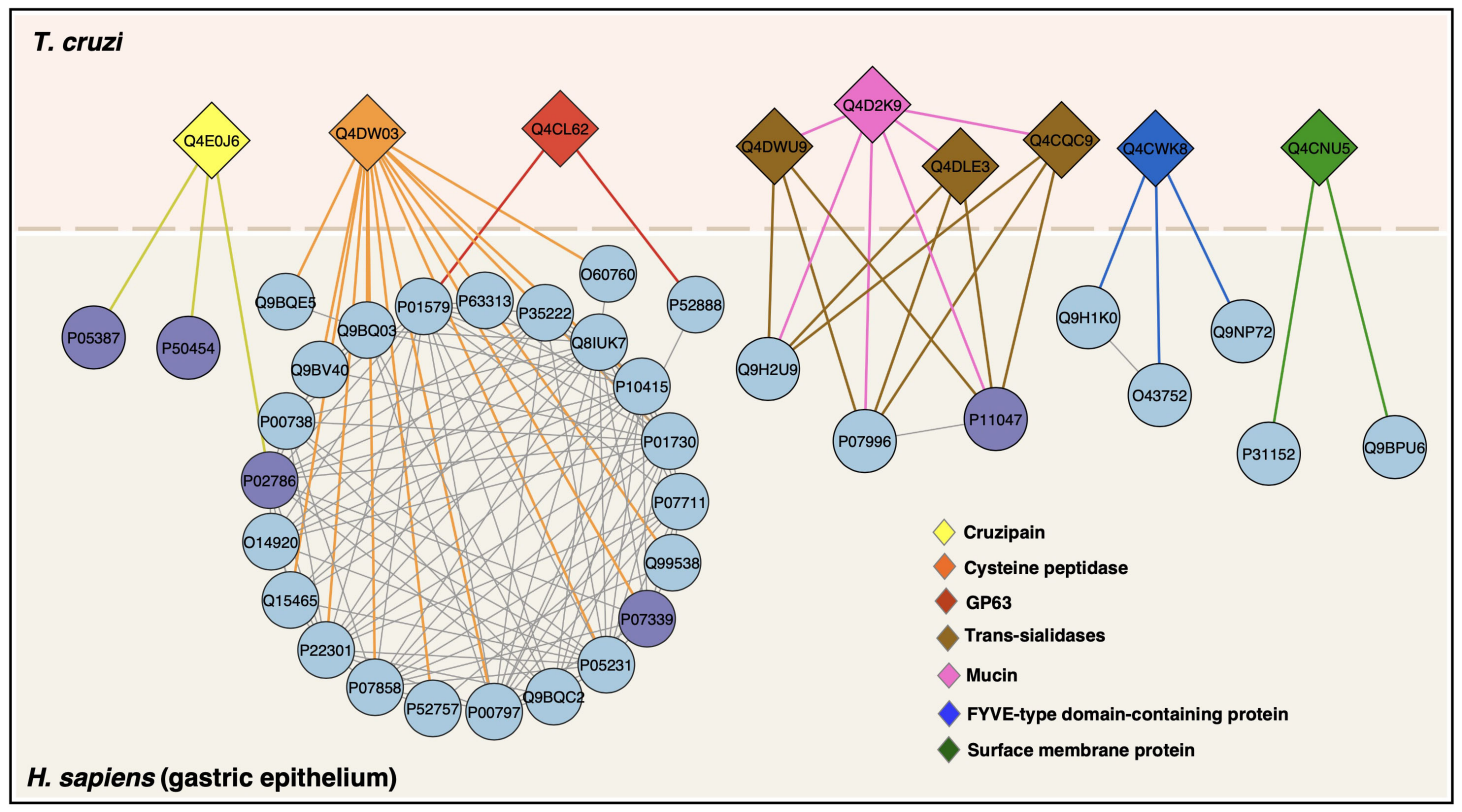
Protein–protein interactions between *T. cruzi* trypomastigotes and gastric epithelium. *T. cruzi* membrane proteins are colored diamonds and human proteins (circles) with predicted interactions are in lightblue and proteins identified by LC-MS/MS in this study are in purple. StringApp 2.0 was used to retrieve and visualize the cross-species network.

Based on protein LC-MS/MS identification and in silico protein-protein interaction (PPI) analysis, we mapped the interaction network of *T. cruzi* membrane proteins and human proteins. Nine proteins were mapped, interacting with 34 human proteins (**Figure 7**). Among the *T. cruzi* proteins are cruzipain, cysteine peptidase, GP63, trans-sialidases, and mucin, among others. Human proteins involved in PPIs such Apolipoprotein L1, Cathepsin D heavy chain, Cathepsin B heavy chain, Catenin beta-1, Serum albumin, T-cell surface glycoprotein CD4 shown a high level of stomach expression level (**Table 2**).

**Table 2:** *Trypanosoma cruzi* (Tc) membrane and human (Hs) proteins mapped with protein-protein interactions and the gene-tissue association for human proteins.

Specie	ID	Protein name	Expression score in stomach tissue
Tc	Q4CL62	GP63	
Tc	Q4DW03	Cysteine peptidase, putative	
Tc	Q4CWK8	FYVE-type domain-containing protein	
Tc	Q4CNU5	Procyclic form surface glycoprotein, putative	
Tc	Q4E0J6	Cruzipain, putative	
Tc	Q4CQC9	Trans-sialidase, putative	
Tc	Q4D2K9	Mucin TcMUCII, putative	
Tc	Q4DLE3	Trans-sialidase, putative	
Tc	Q4DWU9	Trans-sialidase, putative	
Hs	Q9BQ03	Apolipoprotein L1	3.946873
Hs	P07339	Cathepsin D heavy chain	3.925214
Hs	P07858	Cathepsin B heavy chain	3.704597
Hs	P35222	Catenin beta-1	3.634466
Hs	Q8IUK7	Serum albumin	3.495229
Hs	P01730	T-cell surface glycoprotein CD4	3.304488
Hs	P05387	60S acidic ribosomal protein P2	3.273639
Hs	P10415	Apoptosis regulator Bcl-2	3.214685
Hs	P63313	Thymosin beta-10	3.187214
Hs	Q99538	Legumain	3.148305
Hs	P05231	Interleukin-6	3.036941
Hs	P00738	Haptoglobin alpha chain	2.930054
Hs	P01579	Interferon gamma	2.89905
Hs	P22301	Interleukin-10	2.886647
Hs	Q9BQC2	Platelet glycoprotein 4	2.837692
Hs	O14920	Inhibitor of nuclear factor kappa-B kinase subunit beta	2.80289
Hs	P02786	Transferrin receptor protein 1, serum form	2.800633
Hs	Q15465	Sonic hedgehog protein N-product	2.792986
Hs	P07996	Thrombospondin-1	2.715715
Hs	P07711	Cathepsin L1 heavy chain	2.681788
Hs	P50454	Serpin H1	2.609525
Hs	Q9BV40	Vesicle-associated membrane protein 8	2.542898
Hs	O60760	Hematopoietic prostaglandin D synthase	2.364339
Hs	P00797	Renin	2.352538
Hs	P31152	Mitogen-activated protein kinase 4	2.331345
Hs	P11047	Laminin subunit gamma-1	2.088918
Hs	Q9NP72	Ras-related protein Rab-18	2.03159
Hs	Q9H1K0	Rabenosyn-5	1.777921
Hs	O43752	Syntaxin-6	1.477934
Hs	P52888	Thimet oligopeptidase	1.363711
Hs	Q9BQE5	Apolipoprotein L2	1.338456
Hs	Q9BPU6	Dihydropyrimidinase-related protein 5	1.174128
Hs	P52757	Beta-chimaerin	1.090909
Hs	Q9H2U9	Disintegrin and metalloproteinase domain-containing protein 7	0.181818

Tc, *Trypansoma cruzi*; Hs, *Homo sapiens*.

Human proteins are ranked according to their expression score in stomach tissue. The interaction map of these proteins is shown in **Figure 6** and supporting information available in **Supplementary Table 1**.

**Figure 8:**
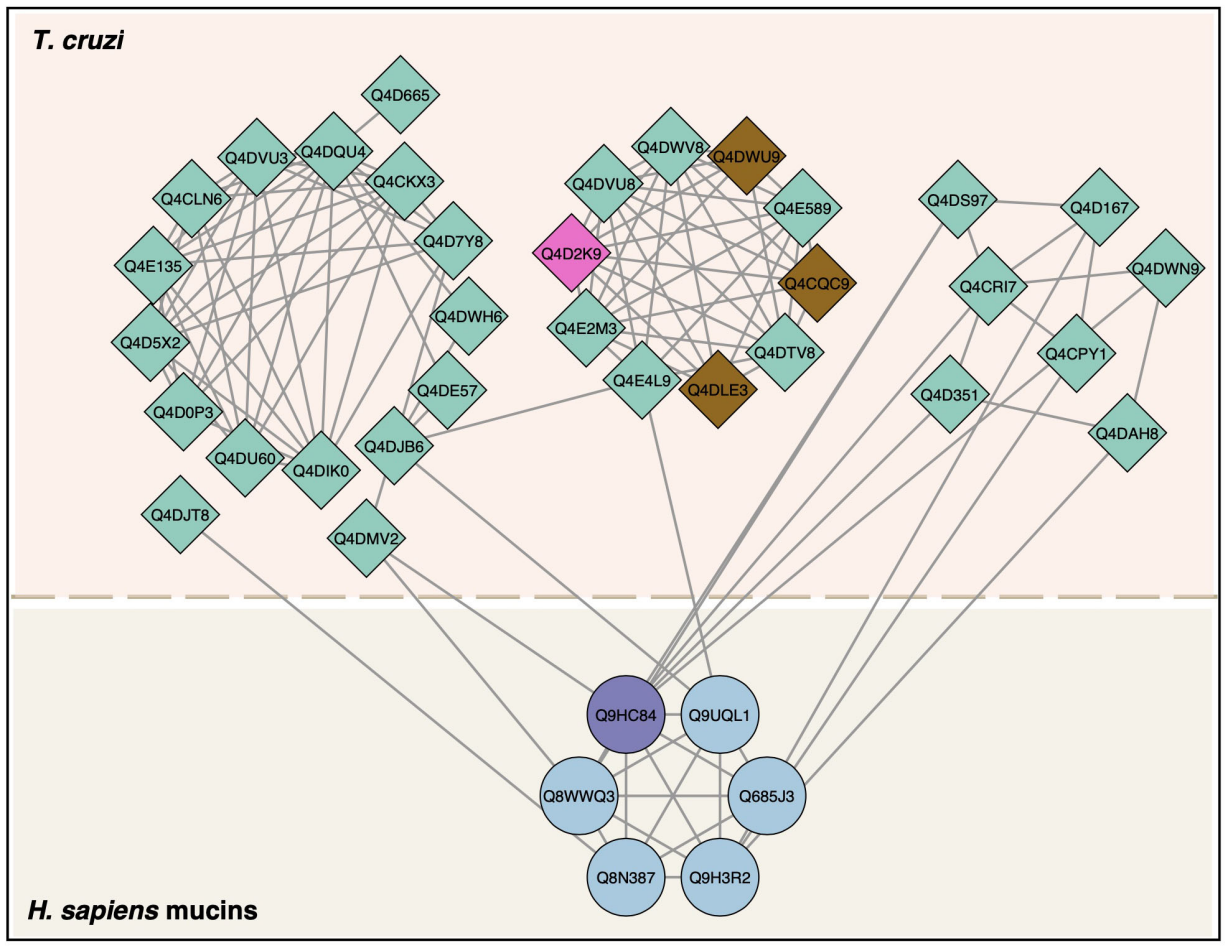
Protein–protein interactions between *T. cruzi* trypomastigote proteins and human mucins. *T. cruzi* proteins (diamonds) and human proteins (circles) interactions were mapped according to StringApp 2.0 database. *T. cruzi* proteins detected by LC-MS/MS in this study were mucin (pink) and trans-sialidase (brown). The human mucin identified by LC-MS/MS in this study was purple.

We then analyzed PPIs between human mucins and *T. cruzi* proteins (**Figure 8**). We found six mucin proteins that interacted with 33 *T. cruzi* proteins. All mucins shown a high level of expression in stomach tissue, except for Mucin-15 (**Table 3**).

**Table 3:** Human (Hs) mucin proteins and *T. cruzi* (Tc) proteins mapped with protein-protein interactions with *T. cruzi* proteins.

Specie	ID	Protein name	Expression score in stomach tissue
Hs	Q8WWQ3	Mucin-5AC	4.57195
Hs	Q9UQL1	Mucin-1 subunit alpha	4.254276
Hs	Q9HC84	Mucin-5B	4.073101
Hs	Q685J3	Mucin-17	3.497127
Hs	Q9H3R2	Mucin-13	3.454218
Hs	Q8N387	Mucin-15	1.628779
Tc	Q4E4L9	Peptidyl-prolyl cis-trans isomerase	
Tc	Q4D665	Palmitoyltransferase	
Tc	Q4D7Y8	ADP-ribosylation factor 1, putative	
Tc	Q4CRI7	60S ribosomal protein L9, putative	
Tc	Q4D351	CMP-sialic acid transporter, putative	
Tc	Q4DWN9	CMP-sialic acid transporter, putative	
Tc	Q4DJT8	Mevalonate kinase	
Tc	Q4DU60	AP-2 complex subunit alpha	
Tc	Q4DTV8	Amidinotransferase	
Tc	Q4DVU8	CRAL-TRIO domain-containing protein	
Tc	Q4DWV8	Glutamate dehydrogenase	
Tc	Q4E2M3	Metaciclina II, putative	
Tc	Q4DWH6	Exportin 1, putative	
Tc	Q4E589	Aminotransferase class IV	
Tc	Q4DS97	Methionine aminopeptidase	
Tc	Q4DAH8	RNA editing ligase, putative	
Tc	Q4DMV2	70 kDa heat shock protein, putative	
Tc	Q4CPY1	60S acidic ribosomal protein P2, putative	
Tc	Q4D167	60S ribosomal protein L19, putative	
Tc	Q4D0P3	ADP-ribosylation factor, putative	
Tc	Q4CLN6	Delta-adaptin, putative	
Tc	Q4DE57	Flagellar radial spoke component, putative	
Tc	Q4CQC9	Trans-sialidase, putative	
Tc	Q4D2K9	Mucin TcMUCII, putative	
Tc	Q4DLE3	Trans-sialidase, putative	
Tc	Q4DWU9	Trans-sialidase, putative	
Tc	Q4E135	Beta-adaptin, putative	
Tc	Q4DJB6	14-3-3 protein, putative	
Tc	Q4CKX3	Clathrin heavy chain, putative	
Tc	Q4DVU3	AP-4 complex subunit epsilon	
Tc	Q4D5X2	Clathrin heavy chain	
Tc	Q4DQU4	Mu-adaptin 1, putative	
Tc	Q4DIK0	AP complex subunit beta	

Human proteins are ranked according to their expression score in stomach tissue. The interaction map of these proteins is shown in **Figure 7** and supporting information are available in **Supplementary Table 1**.

**Figure 9:**
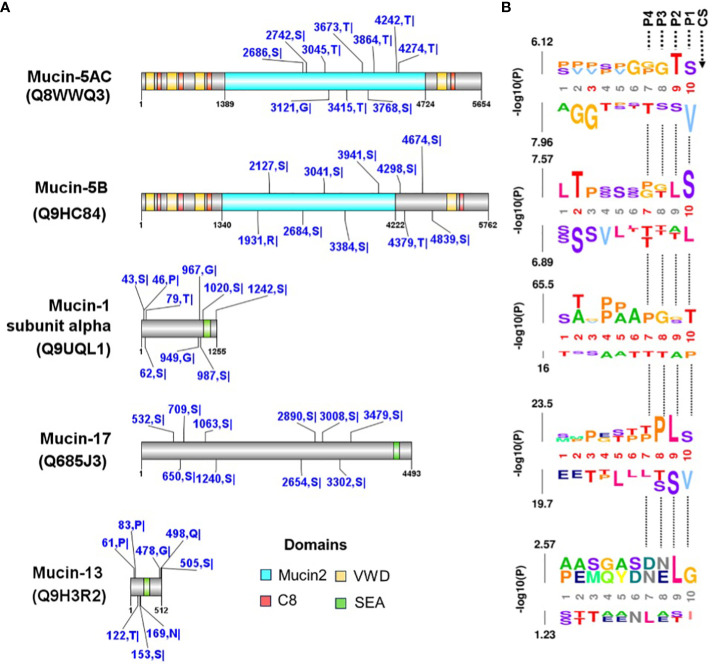
Prediction of cysteine protease cleavage sites in mucin proteins. **(A)** The cleavage sites predicted with a high cut-off for mucins mapped with PPIs between *T. cruzi* (see **Table 3** and **Figure 8**). The top ten scored cleavage sites are shown for each mucin. The domains were identified in the CDD-NCBI conserved domain database, and specific hits are shown. The predicted sites are shown in blue with the amino acid positions and abbreviations. The domains are the Mucin-2 protein WxxW repeating region (this family is a repeating region found on mucins 2 and 5), C8 is a domain that contains eight conserved cysteine residues, VWD is a von Willebrand factor (vWF) type D domain and contains several type D domains, the and SEA domain is proposed to regulate or bind carbohydrate side chains. Recently, proteolytic activity has been demonstrated for the SEA domain. **(B)** The predicted sequence window upstream of the cleavage site was analyzed using the k-mer probability logo (kpLogo) tool for detection and visualization of position-specific amino acid motifs. P1-P4 are the positions of substrate amino acids related to cleavage site (CS) predicted.

Our search for cysteine protease cleavage sites in human mucins using in silico prediction returned tens to thousands of cleavage sites in mucins (**Supplementary Table 2**). We mapped the ten best-ranked sites based on their prediction scores and found that all mucins had this score (**Figure 9A**). Our analysis allowed us to extrapolate the cleavage sites for calpain and cruzipain proteases based on the amino acid motifs present in the P1 and P2 sites of the substrates, in this case in mucins (**Figure 9B**).

**Figure 10:**
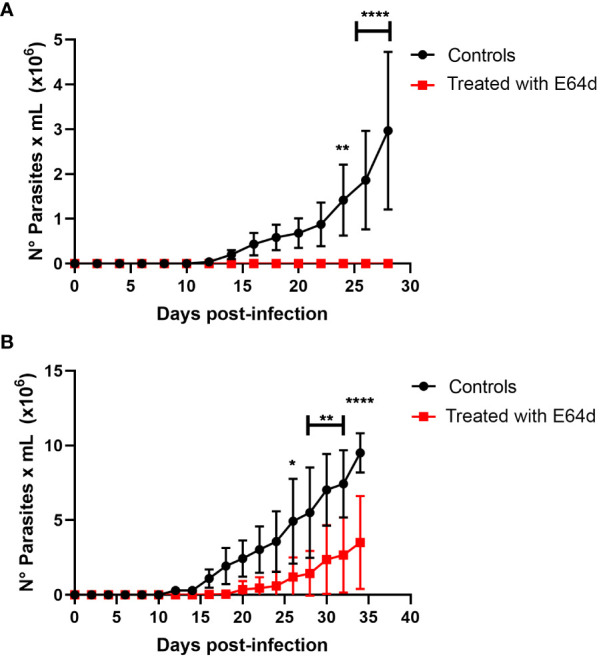
Role of cruzipain in oral *Trypanosoma cruzi* infection. Groups of 5 female Balb/c mice were orally infected using a nasogastric probe with 4x10^5^ trypomastigotes **(A)** or 1x10^5^ amastigotes **(B)** from *T.cruzi* C8C3*hvir* cell line, with or without E 64d cysteine proteinase inhibitor. Parasitemia curve was determined by counting (every 2 days) the number of parasites in a blood sample obtained through a small cut in the tail of the mouse. The results are expressed as the mean ± 1 SD and represent at least three experiments performed in triplicate. **P < 0,01, ***P < 0,001; Two-way ANOVA.

Finally, to corroborate *in vivo* the possible role of cysteine proteinases like cruzipain, the main *T. cruzi* cysteine proteinase seems to play an important role in parasite binding to the stomach cells. We performed oral infection in mice using a nasopharyngeal probe, in which trypomastigotes or amastigotes were treated with or without E 64d (**Figure 10**). Thus, as shown in **Figure 10A**, the infectivity of trypomastigotes previously treated with E 64d was dramatically reduced, while the three mice infected with untreated trypomastigotes developed high parasitemia (**Figure 10A**). In the same way, when amastigotes were pretreated with E64d, parasitemia was significantly decreased (**Figure 10B**).

## Discussion

*T. cruzi* infection is still highly prevalent in Latin America, where active transmission still occurs, especially in Venezuela, Colombia, and Bolivia, where vectors have reappeared (Lidani et al., 2019). However, 70% of the 1,000 cases of acute Chagas disease described in the first 10 years of this century have resulted from oral infections (Shikanai-Yasuda and Carvalho, 2012; Bruneto et al., 2021).

Initially, Chagas disease was endemoenzootic, where the *T.cruzi* cycle in wild environments was maintained in great part by oral infections, when different animals ingested infected vectors or through carnivorism in the prey-predator relationship (Coura et al., 2002; Coura, 2006). However, the latter hypothesis states that it naturally occurs and still occurs in the wild environments of the continent has not been confirmed, and there is no experimental evidence that meat consumption from animals infected with *T. cruzi* can transmit infection. Therefore, this study aimed to demonstrate the role of meat intake from infected animals in the oral transmission of *T. cruzi*.

To approach this question, we fed mice meat from mice in the acute phase of *T. cruzi* infection at the parasitemia peak (**Figure 3A**). Three of the five mice (60%) were infected with *T. cruzi* and died from infection (**Figure 3B**). This observation demonstrated that the intake of infected animal meat could play a role in the oral transmission of *T. cruzi* infection. To our knowledge, this is the first experimental evidence demonstrating the role of carnivorism in Chagas disease transmission. However, Aufderheide et al. (2004) studied 283 mummified bodies from sites in southern Peru and northern Chile and demonstrated a Chagas’ disease prevalence rate of approximately 41% over the past 9,000 years. They suggested that oral transmission might have occurred by ingesting infected, uncooked meat, such as guinea pigs (*Cavia porcellus*). If the raw meat from *T.cruzi* infected mice was used to infect the mice that were consumed, trypomastigotes or intracellular amastigotes could be responsible for the infection. Experimental infections were performed using different inoculums at these two parasite stages to investigate this.

When mice were infected with a nasogastric probe with different amastigotes inoculums, all animals were infected (**Figure 4**). One hundred percent of the mice were infected with an inoculum of 3×10^5^ (**Figure 4B**), whereas 80% were infected with an inoculum of 2×10^5^ (**Figure 4C**). Moreover, 60% of the mice were infected with the inoculum of 1x10^5^ (**Figure 4D**). Similarly, trypomastigotes were infective when inoculums of 4 × 10^5^ were used, resulting in four of the five infected mice (80%) (**Figure 5A**). When inoculums of 1 × 10^5^ were used, 4 of 5 mice inoculated (80%) were also infected (**Figure 5B**). This evidence shows that both, TCTs and amastigotes can invade the gastric mucosa and cause oral infections. To our knowledge, the role of amastigotes in oral transmission of *T. cruzi* has not been evaluated. However, unlike metacyclics trypomastigotes (MT), it has been proposed that blood trypomastigotes are inefficient in infecting mice via the oral route (Hoft, 1996). The reason for these differences in infectivity between these two stages is not well known but could be due to the migration capacity of the two infectious forms through the gastric mucin layer (Cortez et al., 2012). When tested for the possible use of TCTs, it was found that the parasite traversed the mucin layer at a two-fold lower number than metacyclic trypomastigote (MT). The possibility of blood trypomastigotes being more susceptible to pepsin digestion at an acidic pH has also been proposed (Cortez et al., 2006). In our study, we observed that TCTs from the C8C3 ***hvir*** cell line were highly susceptible to pepsin lysis. This susceptibility and reduced ability to pass through mucin may explain why not all mice were infected. Similarly, differences in parasitemia peaks with respect to MT infections were observed. How can this TCTs delay the peak of parasitemia be explained? One of the most likely explanations could be based on the fact that MTs are highly resistant to degradation by pepsin (Cortez et al., 2012)). Therefore, the total inoculum administered can be used to infect stomach host cells. However, our experiments on migration through mucin in the presence or absence of pepsin at acidic pH and the *in vitro* infection of AGS cells in the presence of mucin showed that the situation with TCTs is completely different from that with MTs. Only a small number of TCTs cross mucin and can resist pepsin and acidic pH. Therefore, if we extrapolate this condition to what could occur *in vivo*, it is possible to hypothesize that only a very small portion of the TCTs inoculum would survive and invade the stomach cells. Since there is a small amount of infecting inoculum, the development of the infectious process would be delayed; therefore, the parasitemia peak would also be delayed.

However, we demonstrated that TCTs from the virulent cell line C8C3*hvir* and amastigotes were highly infectious in mice. Nevertheless, the origin of the observed differences in the infectivity of blood trypomastigotes and TCTs may also be due to their genetic background. Little information is available regarding the genotypes associated with oral *T. cruzi* infections. However, DTU 1 seems to be the most frequently encountered, where haplotipes TcId has been identified in *T cruzi* isolates from rats, triatomines, and patients (Marcili et al., 2009; Andrade et al., 2011; Ramírez et al., 2013; Díaz-Bello et al., 2014). As the C8C3 ***hvir*** cell line corresponds to DTU 1, this may partly explain its ability to cause oral infections. Nevertheless, studies of metacyclic forms of different strains have suggested that they are poorly infective (Maeda et al., 2016). However, our evidence using TCTs suggests otherwise. Thus, it is possible to theorize that DTU 1 could be poorly infective if transmitted by contamination with triatomine feces containing metacyclic forms of the parasite but may be highly infective when consumed in meat from infected animals having blood trypomastigotes or amastigotes.

If trypomastigotes and amastigotes are infectives by the oral route, we raise the question of which molecules could be involved in parasite binding to mammalian stomach cells. When the immunoblots of AGS cells incubated with amastigote cell lysates were incubated with different antibodies, only antibodies raised against cruzipain and the Ssp-4 epitope, recognized by the monoclonal antibody 2C2, were detected (**Figure 6A**). In trials with trypomastigote lysates, we observed that only antibodies against cruzipain and transialidase were able to detect the proteins associated with AGS cells (**Figure 6B**), whereas immunoblots in which antibodies against CRP or gp85 did not detect reactivity.

A shaving experiment was performed to confirm these observations, and proteins associated with the surface of human stomach AGS cells were identified by LC-MS/MS (**Table 1**). Among the *T. cruzi* TCT membrane proteins identified, several trans-sialidases were observed in addition to cysteinyl proteinases and Gp63. The presence of trans-sialidase and cysteine proteinases was consistent with our observations derived from the western blot assays. Gp63 was not studied by western blotting. However, the role of Gp63 in *T. cruzi* infectivity has been suggested by Cuevas et al. (2003). On the other hand, trans-sialidase is a unique *T. cruzi* enzyme involved in transferring sialic acid from host glycoconjugates to mucins (Eugenia Giorgi and De Lederkremer, 2011). TS is involved in several virulence mechanisms, including invasion and host cell recognition (Magdesian et al., 2007; Dc-Rubin and Schenkman, 2012; Freire-de-Lima et al., 2012). The presence of sialylated epitopes on the surface of infective forms of *T. cruzi* increases infectivity (Piras et al., 1987; Schenkman et al., 1991). Similarly, sialylation and desialylation of the host cell surface can modulate parasite adherence and penetration (Freire-de-Lima et al., 2012). Therefore, it is possible to hypothesize that stomach mucus could be a source of sialic acid that positively regulates the invasion of the stomach epithelium through parasite TS activity. Considering that among the molecules detected both in the blots and in the shaving experiment associated with LC-MS/MS, we identified cruzipain and cysteine proteases, we decided to deepen our knowledge regarding the potential role of this protease in oral *T. cruzi* infection.

We then performed oral infection in mice, in which trypomastigotes or amastigotes treated with or without E64d were used to infect the mice (**Figure 10**). As shown in **Figure 10A**, the infectivity of trypomastigotes previously treated with E64d was dramatically reduced, whereas three mice infected with untreated trypomastigotes developed high parasitemia (**Figure 10A**). In contrast, parasitemia significantly decreased when amastigotes were pretreated with E64d (**Figure 10B**). These results suggest that cruzipain, the principal *T. cruzi* cysteinyl proteinases (and other parasite cysteine proteinases), plays an important role in parasite association with stomach cells. According to electron microscopy studies, Czp is located in the flagellar pocket of the TCTs being the main secreted lysosomal peptidase in *T. cruzi* (Souto-Padrón et al., 1990). Several studies using synthetic irreversible cysteine peptidase inhibitors have demonstrated that *T. cruzi* infection, host immune evasion, and intracellular growth depend on Czp activity (Meirelles et al., 1992; Tomas et al., 1997; Scharfstein et al., 2000; McKerrow et al., 2008; Doyle et al., 2011). Pretreatment of TCTs of the C8C3 ***hvir*** cell line with E64d has been shown to significantly inhibit their infectivity both *in vivo* and *in vitro* (San Francisco et al., 2017). In the stomach, the gastric mucosa is the first natural barrier responsible for protecting the host against internal insults, such as the acidic pH of gastric juice, and external insults, such as microorganisms (Deng et al., 2021). Thus, cruzipain and other cysteinyl proteinases may play a critical role in the invasion of TCTs into the gastric mucosa. Although the molecular mechanisms involved in this process are unknown, it is possible to speculate that these proteases could participate in mucus degradation, an event before parasite contact with stomach epithelial cells. The participation of cysteinyl proteinases in the degradation of gastric mucus before parasite contact with the intestinal epithelium has been described in *Entamoeba histolytica* (Lidell et al., 2006).

In MT, it has been described that the surface glycoprotein gp82 plays a central role in establishing *T. cruzi* infection in mice via the oral route (Yoshida, 2008). gp82 is critical for oral *T. cruzi* infection because it binds gastric mucin in the mucus layer, which protects the stomach mucosa (Staquicini et al., 2010). It has been proposed that upon binding to gastric mucin, MT migrates through the mucus layer and reaches the underlying epithelial cells, invading in a gp82-mediated manner (Neira et al., 2003; Cortez et al., 2006; Covarrubias et al., 2007). However, no trypomastigote molecules have been reported to play a role in bloodstream trypmomastigotes or their counterparts in TCTs.

Here, we observed that cruzipain and transialidase were involved in parasite binding to stomach cells (**Figure 6**). In contrast, cruzipain digests fibronectin (FN). The interaction between fibronectin and *T. cruzi* has also been previously described. Experiments with TCT revealed the involvement of fibronectin in target cell adhesion/invasion (Ouaissi et al., 1985). Using *T. cruzi* CL strain metacyclic forms, it was reported that fibronectin present in the cell culture medium bound to metacyclic forms is digested by cruzipain. Treating HeLa cells with purified recombinant cruzipain increased parasite internalization, whereas treatment with cysteinyl proteinase inhibitors had the opposite effect. This evidence confirms that the cysteine proteinase of *T. cruzi* metacyclic forms, through its fibronectin-degrading activity, is involved in host cell invasion (Maeda et al., 2014). We speculated that cruzipain acts on stomach cells to degrade fibronectin and other glycoproteins in the extracellular matrix and promotes parasite invasion.

Several pieces of evidence strongly suggest that C8C3 ***hvir*** virulence is related to the differential expression of well-known virulence factors, including Czp and TS (San Francisco et al., 2017). In addition, parasite treatment with E64d strongly reduced parasite oral infection capacity and parasitemia levels in BALB/c mice. Thus, the concerted action of cysteinyl proteinases and TS could contribute to the success of TCTs in the invasion of the gastric mucosa. Although the molecule that binds gastric mucins is unknown, trans-sialidase could act on the O-linked mucins present in the mucous layer of the stomach, removing sialic acid and sialydating the parasite membrane, making it more invasive. On the other hand, cysteinyl proteinases could degrade the gastric mucins, favoring the progression of TCTs through the mucous layer to finally access the stomach epithelium, where the classical mechanisms of invasion could operate.

Our in silico analysis of PPIs allowed us to identify important *T. cruzi* proteins involved in interactions with host proteins, including trans-sialidases and cruzipain, as well as proteins identified by LC-MS/MS in our proteomic dataset. Furthermore, these proteins interact with human proteins with high expression in stomach tissue, as seen in the gene-tissue association analysis.

Our interaction analysis involving host mucins, despite having demonstrated interaction with gp85 or gp82, nevertheless demonstrated that human mucins interact with *T. cruzi* protein complexes that are related to the transfer of sialic acid, such as the CMP-sialic acid transporter, putative, and trans-sialidase itself.

Since the activity of cysteine proteases is important in host cell interaction and invasion, we predicted substrate cleavage sites for cysteine proteases in mucin proteins, specifically for calpains, an important family of Ca^+2^-dependent cysteine proteases that contain a nucleophilic cysteine in the catalytically active site. The expression and cellular localization of *T. cruzi* calpains revealed that are distributed in different cellular components in all life cycle forms of the parasite, including plasma membrane (Ennes-Vidal et al., 2020) and thus, if calpains cleave mucins, they can regulate the interaction of the parasite in stomach cells. Thus, we predicted the calpain cleavage sites in mucins that were mapped in the interaction network with *T. cruzi* proteins. Site prediction showed that mucins have several cleavage sites for the cysteine protease calpains (**Figure 9A**). Another important family of cysteine proteases are cathepsins, which include cruzipain. The studies on substrate profiling of cysteine proteases showed that cruzipain has a preference for hydrophobic residues (such as Leucine and Glycine) in the P2 position of the substrates (Choe et al., 2006; Dos Reis et al., 2006; Ren et al., 2009; Wang et al., 2023). Our k-mer probability logo analysis of sequence window upstream predicted sites (**Figure 9B**) corroborates these previous results for cruzipain and reinforces the potential relationship of *T. cruzi* cysteine proteases, both calpain and cruzipains, with the invasion of stomach cells.

In summary, it was concluded that, under our experimental conditions, consuming meat from infected animals in the acute phase allows the transmission of *T. cruzi* infection. However, the possibility that oral transmission may be feasible when animals are in the indeterminate or chronic phase cannot be ruled out. At these stages of *T. cruzi* infection, the success of oral transmission may depend on the organ and tissue parasite load and the genetic haplotype of *T. cruzi*, which could determine its ability to resist acidic pH, cross mucins, and infect gastric cells. Thus, it is possible to postulate that the DTUs involved may be critical to the success of oral infection. Indeed, Hoft et al. (1996) reported poor infectivity of blood trypomastigotes in Tulahuen strain (TC VI). On the other hand, Cortez et al. 2012, also suggested that trypomastigotes were poorly infective but used Y strain (Tc II). In addition, the intrinsic virulence of each strain or isolate, indicated by a higher expression of virulence factors such as Cz, TS, CRP, and gp63, among others, should be considered (Osorio et al., 2012). Furthermore, upregulation of bioenergetic and biosynthetic pathways has been proposed as a new aspect to be considered in *T. cruzi* virulence (San Francisco et al. 2022). In addition, the genetic background of the host should also be considered, and a strain or isolate of a given DTU that is infective for one species might not be infective for another.

Transmission via the oral route is an emerging scenario in Chagas disease. This transmission mechanism displays a habitual character in the primitive and endemic cycle of the parasite, which occurs through consuming contaminated food and/or beverages by dejection of triatomine feces (PAHO/WHO, 2022). Here, we experimentally demonstrate that carnivorism is a mechanism of *T. cruzi* transmission. Although this is perhaps the major mechanism by which parasite infection is transmitted in the wild, the possibility of human transmission cannot be ruled out. In endemic areas where peridomestic and wild vectors are present concomitantly with rabbits, guinea pigs, lambs, and others, the meat of infected animals could be a source of human infection. Therefore, sanitary education of the population is necessary to prevent the ingestion of raw meat and to encourage the use of previously frozen or cooked meat. Therefore, if Chagas disease is a food-borne infection, implementing prophylactic measures, such as sanitization, freezing, or using heat, accompanied by qPCR detection methods, should be considered (de Oliveira et al., 2019). Furthermore, implementing health education programs for populations living in endemic areas of potential risk from the perspective of oral transmission of Chagas disease and the implementation of traveler advisories for tourists visiting these areas is an absolute necessity. These traveler advisories should emphasize avoiding the consumption of tropical fruit juices, pulps, and exotic meats (Franco-Paredes et al., 2020; López-García and Gilabert, 2023). Thus, considering that oral transmission of Chagas disease involves animals, the environment, and humans, only a concerted strategy under the One Health concept would succeed in controlling this form of transmission of this parasitic infection (López-García and Gilabert, 2023).

Finally, experimental studies will be necessary to define the differences observed in the infectivity of blood trypomastigotes or their equivalents, the TCTs. These should be oriented to define whether their infectivity depends on the genetic background of the parasite, on the infected species and its genetic background, or both.

## Data availability statement

The datasets presented in this study can be found in online repositories. The names of the repository/repositories and accession number(s) can be found in the article/**Supplementary Material**.

## Ethics statement

The animal study was approved by Comité de Ética de la Investigación Científica de la Universidad de Antofagasta. The study was conducted in accordance with the local legislation and institutional requirements.

## Author contributions

VT: Conceptualization, Investigation, Writing – original draft, Writing – review & editing, Data curation, Formal analysis, Methodology, Validation. VC: Investigation, Data curation, Formal analysis. BG: Data curation, Investigation, Methodology, Writing – review & editing, Software, Supervision. JSF: Investigation, Methodology, Data curation, Software, Supervision, Writing – review & editing. AC: Data curation, Investigation, Software, Formal analysis, Resources, Writing – review & editing. JLV: Data curation, Investigation, Methodology, Formal analysis, Funding acquisition, Resources, Writing – review & editing. KM: Data curation, Investigation, Resources, Software, Methodology, Writing – original draft. LJF: Data curation, Formal analysis, Funding acquisition, Methodology, Resources, Writing – review & editing, Conceptualization, Software. RdA: Bioinformatic analysis, Writing-review and edition. AK: Funding acquisition, Writing – review & editing. JG: Conceptualization, Funding acquisition, Resources, Writing – review & editing, Investigation, Supervision, Visualization, Writing – original draft.
