# Supplementary figures and images for: Oral infectivity through carnivorism in murine model of Trypanosoma cruzi infection
Source: Front Cell Infect Microbiol. 2024 Feb 22;14:1297099. doi: 10.3389/fcimb.2024.1297099 (PMC10941204; doi:10.3389/fcimb.2024.1297099)

Supplementary figures

Figure S1

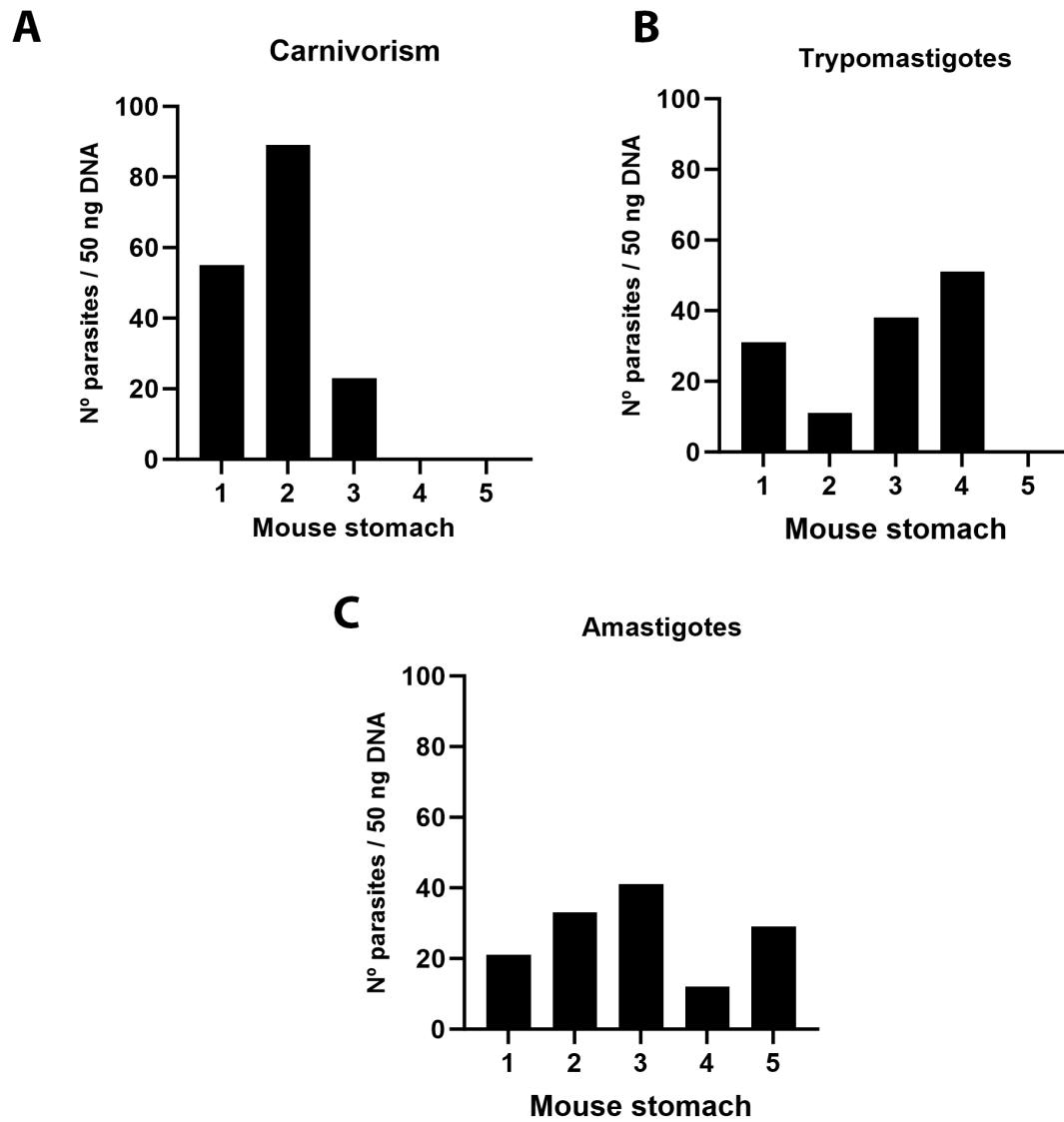

Supplement: Supplementary file 3 [file Presentation_1.pdf]
